# Research Knowledge Progression in Allied Health Sciences Students: A Cross‐Sectional Study

**DOI:** 10.1002/hsr2.72935

**Published:** 2026-07-29

**Authors:** Meera Indracanti, Aparna Srikantam, Mequanente Dagnaw

**Affiliations:** ^1^ School of Allied and Healthcare Sciences Malla Reddy University Hyderabad Telangana India; ^2^ Department of Epidemiology and Biostatistics, Institute of Public Health College of Medicine and Health Sciences, University of Gondar Gondar Ethiopia; ^3^ Department of Medical Biotechnology, Institute of Biotechnology University of Gondar Gondar Ethiopia

**Keywords:** allied health education, competency‐based education, India, research education, research participation, undergraduate students

## Abstract

**Background:**

Research education is fundamental to evidence‐based practice in the allied health sciences; however, the progression of students from basic awareness to applied research competency remains poorly characterized. Understanding these progression patterns is essential for curriculum development and educational reform.

**Methods:**

We conducted a cross‐sectional survey of 730 undergraduate students selected through stratified convenience sampling from 10 allied health science programs at Malla Reddy University, Hyderabad, India. Data were collected using a structured, self‐administered questionnaire developed through literature review, expert consultation, and pilot testing. Research competencies were measured across four sequential domains research awareness, definitional knowledge, methodological familiarity, and practical experience using binary (Yes/No) items designed to capture progression from basic understanding to active research engagement. Data were analyzed using chi‐square tests, correlation analysis, and multiple regression modeling. Key methodological limitations included the single‐institution setting, self‐reported responses, and the cross‐sectional design, which may limit generalizability and preclude causal inferences.

**Results:**

A progressive decline in competency was observed across learning domains: 74.9% demonstrated basic research awareness, 66.3% could define research concepts, 36.2% understood research methodology, and only 26.7% had practical research experience (*p* < 0.001). This represents a 48.2 percentage point decline from awareness to application. Research aptitude scores remained consistent across all academic programs (χ^2^ = 0.247, *p* = 0.999). Students with formal research training demonstrated significantly higher participation rates (AOR = 2.85, 95% CI: 1.98–4.11, *p* < 0.001).

**Conclusion:**

While allied health curricula successfully establish foundational research awareness, they often fail to support progression to practical application. Educational interventions that incorporate active learning methodologies, structured mentorship, and experiential research opportunities are necessary to bridge the theory‐practice gap.

## 1 Introduction

Research education has become a fundamental component of allied health sciences curricula worldwide, playing a critical role in preparing students to become evidence‐based practitioners capable of integrating scientific knowledge into clinical decision‐making. Competency in research not only enhances critical thinking and problem‐solving skills but also supports lifelong learning and improves healthcare outcomes [1]. Despite its recognized importance, substantial gaps persist in the integration and effectiveness of research training within undergraduate health sciences education. A recent systematic review by King et al. highlighted that many students graduate with inadequate preparation for research engagement, particularly in areas such as study design, data interpretation, and critical appraisal [2]. Similarly, consistently low levels of research preparedness across institutions, with students frequently expressing limited confidence in their methodological knowledge and application skills [3].

In the Indian context, the integration of research into undergraduate curricula faces additional structural and contextual challenges. Studies have identified barriers such as curriculum overload, limited dedicated time for research training, inadequate mentorship, and insufficient faculty capacity to support research activities [4]. These constraints are further compounded by motivational and attitudinal factors among students, including low perceived relevance of research and limited exposure to hands‐on research experiences, as noted in health professions education literature (Wynants et al., 2020). Consequently, while the importance of research education is widely acknowledged, its implementation remains inconsistent, and students often progress through their training without achieving meaningful research competency [5].

Existing literature has largely focused on assessing overall research knowledge or attitudes; however, there is a lack of empirical evidence examining how research competency develops progressively across different stages of undergraduate education, particularly in allied health sciences. Educational frameworks such as Bloom's Taxonomy and Miller's Pyramid suggest that learning evolves from basic awareness and understanding to higher levels of application and performance [6]. Yet, few studies have operationalized these frameworks to map the progression of research knowledge from foundational awareness to practical engagement in real‐world settings. This gap is especially evident in low‐ and middle‐income countries, where contextual constraints may further influence learning trajectories. To address this gap, the present study provides a comprehensive assessment of research knowledge progression among undergraduate allied health sciences students in India. Specifically, we examined four key competency levels basic awareness, definitional knowledge, methodological familiarity, and practical experience and explored factors associated with their development across academic years and programs. We hypothesize that research knowledge increases with academic progression and is positively associated with exposure to research training and participation in research‐related activities.

## 2 Theoretical Framework

Our investigation builds on two complementary theoretical foundations that explain the progression of student learning. Constructivist learning theory suggests that knowledge develops through progressive elaboration, where learners actively build upon foundational concepts to develop increasingly complex applications. Carraccio et al. demonstrated that structured competency‐based education frameworks emphasize sequential skill development through clearly defined learning outcomes [7].

**Figure 1:**
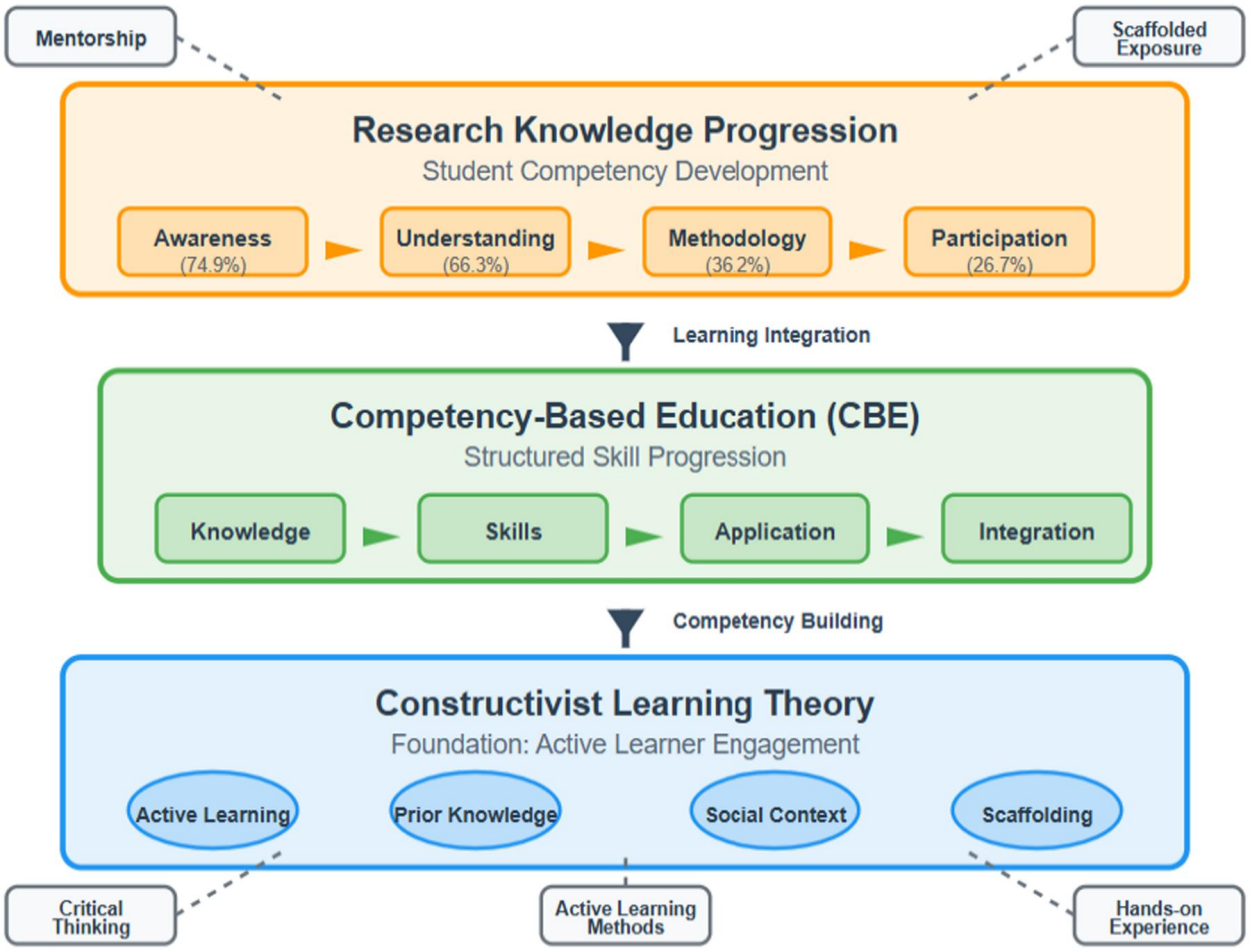
Theoretical learning framework guiding research knowledge progression.

Competency‐based education (CBE) frameworks emphasize the sequential acquisition of knowledge, skills, and attitudes, rather than time‐based progression. Frenk et al. highlighted that CBE provides transparency in training approaches and ensures systematic development of competency [8]. Oostra et al. further showed how integrating competency‐based education with problem‐based learning can enhance student engagement and practical skill development in health sciences courses [8]. See Figure 1 for a schematic of the theoretical learning framework that guided our analysis.

According to this framework, students should progress through distinct competency levels, with each building upon the previous stage. However, whether this progression occurs naturally or uniformly across student populations has rarely been tested empirically in health sciences education.

## 3 Research Gaps and Study Context

Previous medical education studies have identified declining competency patterns across various learning domains, but these findings have not been systematically examined in allied health sciences programs. Ashour and Hatamleh found that allied health professionals often lack research preparedness due to insufficient undergraduate training, particularly in methodological knowledge and practical research skills [9].

The Indian higher education context adds additional complexity. Beyond the barriers identified by Unnikrishnan et al., Indian institutions face resource constraints and pedagogical challenges that may affect research competency development. Motivational and preparedness issues further complicate this picture, as documented by Wynants et al. in their study of undergraduate nursing students [10].

To address these gaps, this study examined the progression of research knowledge among undergraduate students in allied health sciences in India across four key competency levels: basic research awareness, definitional knowledge, methodological familiarity, and practical experience. Specifically, the study aimed to assess how research competencies progress across different learning domains, identify factors associated with higher research aptitude, and determine whether progression patterns vary across different allied health programs. The study hypothesized that research competencies would demonstrate a sequential progression from awareness to practical experience, that students with greater exposure to research‐related learning opportunities would exhibit higher research aptitude, and that significant differences in competency progression would exist across allied health disciplines.

## 4 Methods

### 4.1 Study Design and Setting

A cross‐sectional survey was carried out among undergraduate students enrolled in the School of Allied and Healthcare Sciences at Malla Reddy University, Hyderabad, India, during the January 2023 to December 2024 academic year. The institution offers 10 allied health science programs, ensuring broad representation across diverse disciplines within the field.

### 4.2 Sample Size Determination

The minimum required sample size was initially calculated using the single population proportion formula with a 95% confidence interval, 5% margin of error, and an assumed proportion (*p* = 50%) to maximize sample variability in the absence of prior comparable data. This yielded a preliminary sample size of 384 participants. Because the total source population was finite (*N* = 730), a finite population correction was applied, reducing the required sample size to 252. After adding a 10% allowance for potential non‐response, the final estimated minimum sample size was 277 participants. However, to improve representativeness, minimize sampling error, and strengthen the statistical power of subgroup analyzes across academic programs and years of study, a census approach was adopted in which all 730 eligible undergraduate students were invited to participate.

### 4.3 Participants and Sampling

The study population comprised 730 undergraduate students across all years of study, from first year to final year. Participants were recruited using a stratified convenience sampling approach from 10 allied health sciences programs: Anesthesia and Operation Theater Technology (AOTT), Life Sciences (BMB), Cardiovascular Technology (CVT), Medical Biotechnology (MBT), Bachelor of Physiotherapy (BPT), Optometry, Emergency Medicine and Critical Care Technology (EMCCT), Radiology and Imaging Technology (RIT), Clinical Nutrition and Dietetics (CND), and Medical Laboratory Technology (MLT). Strata were defined according to academic program and year of study to ensure representation across disciplines and levels of training. Within each stratum, all eligible students who were present during the data collection period were invited to participate using a convenience sampling approach.

Students were eligible if they were actively enrolled in an allied health sciences program and willing to participate. Those absent during data collection or who declined participation were excluded. A total of 730 students participated, yielding a 100% response rate among approached individuals, indicating strong engagement across programs. However, because participant selection within strata was based on availability rather than random sampling, the possibility of selection bias cannot be excluded, which may limit the generalizability of the findings.

## 5 Variables

### 5.1 Dependent Variable

Research knowledge progression among allied health sciences students (measured through levels of research awareness, definitional knowledge, methodological familiarity, and practical research experience).

### 5.2 Independent Variables

The independent variables included students' allied health program or discipline, year of study, age, gender, previous exposure to or participation in research activities, attendance at research workshops or seminars, access to research mentorship or guidance, and academic background or performance. These variables were examined to determine their association with research knowledge progression among allied health sciences students.

## 6 Operational Definitions

**Basic awareness**: Defined as recognition of the concept and general purpose of research, corresponding to the lowest level of cognitive learning (“knowledge/remembering”) in Bloom's Taxonomy [11].

**Definitional knowledge**: Defined as the ability to articulate a basic definition of a research study, reflecting conceptual understanding (“understanding” level in Bloom's framework) [12].

**Methodological familiarity**: Defined as self‐reported understanding of basic research methods and study designs, representing applied knowledge (“knows how”) within Miller's Pyramid of Competence [13].

**Practical experience**: Defined as direct participation in research‐related activities, reflecting the highest level of competence (“does”) in Miller's framework and experiential learning theory [13, 14].

### 6.1 Justification of Cutoffs and Scaling of Research Competency

The use of binary (Yes/No) response options and corresponding cutoffs for each competency level was based on the study objective of identifying whether students had achieved essential milestones in research competency progression rather than measuring the degree of proficiency. Each competency domain research awareness, definitional knowledge, methodological familiarity, and practical experience was operationalized using clearly defined indicators derived from literature review, expert consultation, and pilot testing. A “Yes” response indicated attainment of the minimum expected competency within that domain, while a “No” response indicated lack of attainment. This binary scaling approach was selected because no validated multidimensional scale was available for assessing staged research competency progression among allied health sciences students in the local context. The simplified scaling enhanced interpretability, facilitated classification across sequential competency levels, minimized respondent burden, and improved feasibility for large classroom‐based data collection. Additionally, binary measures are commonly applied in exploratory educational and competency‐based research where the primary aim is to determine competency attainment status rather than quantify intensity or advanced skill differentiation [15].

The scaling of research competency into four hierarchical levels basic awareness, definitional knowledge, methodological familiarity, and practical experience was theoretically grounded in established educational frameworks. Bloom's Taxonomy conceptualizes learning as progressing from lower‐order cognitive processes (remembering and understanding) to higher‐order application [16]. Accordingly, basic awareness corresponds to “remembering,” while definitional knowledge reflects “understanding.” Miller's Pyramid further supports this progression in terms of competence, moving from “knows” and “knows how” to “shows how” and “does.” In this study, methodological familiarity aligns with applied understanding (“shows how”), and practical experience represents real‐world performance (“does”). Additionally, Kolb's Experiential Learning Theory emphasizes that active participation is essential for deeper learning, supporting the inclusion of practical experience as the highest level of competency.

Together, these frameworks justify the ordinal structure of the competency scale as a progression from cognitive awareness to experiential engagement. While the binary cutoffs simplify complex constructs and may limit sensitivity, they are appropriate for capturing stage‐wise attainment in an exploratory cross‐sectional design. Future research should consider developing multi‐item, scaled instruments to capture gradations in competency and allow for more robust psychometric evaluation [13, 17].

### 6.2 Conceptual Justification

The classification of research competency into hierarchical levels is grounded in established educational theories. Bloom's Taxonomy conceptualizes learning as progressing from basic knowledge and comprehension to application and higher‐order skills. Similarly, Miller's Pyramid of Clinical Competence describes progression from “knows” and “knows how” to “shows how” and “does.” In this study, basic awareness corresponds to “knows,” definitional knowledge to “knows how,” methodological familiarity to applied understanding (“shows how”), and practical experience to real‐world performance (“does”) [12, 13].

Furthermore, competency‐based education frameworks emphasize the progressive acquisition of knowledge, skills, and practice, aligning with the structure used in this study. Although the measures are based on self‐report and simplified binary responses, they provide a pragmatic approach to capturing stages of research competency in large student populations [7, 8].

### 6.3 Data Collection

The survey instrument was developed through an iterative process involving literature review, expert consultation, and pilot testing, guided by established principles of competency‐based curriculum design [7, 8]. The measurement of research competency in this study was informed by established theories of competency‐based education and learning progression. Frameworks such as Miller's Pyramid of Clinical Competence and Bloom's Taxonomy conceptualize knowledge development as a continuum from basic awareness to higher‐order application. Accordingly, basic awareness reflects recognition of the concept of research (“knows”), definitional knowledge represents the ability to articulate core concepts (“knows how”), methodological familiarity reflects understanding of research methods and study designs (“shows how”), and practical experience captures engagement in research activities (“does”). This hierarchical structure aligns with competency‐based education models emphasizing progressive acquisition of knowledge, skills, and practice [7, 8].

The questionnaire items were developed based on literature review, expert input, and pilot testing; however, they do not constitute a previously standardized or fully validated scale. Reliability testing was not conducted, and pilot testing results were not formally analyzed or reported. Therefore, the measures should be considered pragmatic and exploratory, reflecting perceived competency rather than a comprehensive or psychometrically robust assessment. The use of binary (Yes/No) items further simplifies complex constructs and may limit measurement sensitivity. For participants reporting practical experience, the duration of involvement was additionally recorded in months. The questionnaire was administered in hard‐copy format between September and November 2023. Future studies are recommended to employ validated, multi‐item instruments with established reliability and systematically evaluated pilot findings to strengthen measurement rigor.

Several measures were taken to minimize potential biases. To reduce selection bias associated with convenience sampling, a stratified approach was employed based on academic program and year of study, ensuring representation across disciplines and training levels. Within each stratum, all eligible and available students were invited to participate, which helped improve coverage of the target population. To limit information bias arising from self‐reported data, a standardized, structured questionnaire with clearly worded items was used, and pilot testing was conducted to enhance clarity and consistency of responses. Data collection procedures were uniform across participants. To mitigate social desirability bias, participants were assured of anonymity and confidentiality, and no personal identifiers were collected. Data were collected using structured self‐administered hard‐copy questionnaires administered in the absence of instructors or supervisors to encourage honest and unbiased responses.

### 6.4 Composite Score Development

We constructed three composite indices to provide a comprehensive assessment of research competency. The Research Aptitude Score (0–100 scale) combined responses from four knowledge questions (scored 0–1 each) and research confidence ratings (1–5 scale). We calculated scores as: (Raw Score ÷ Maximum Possible Score) × 100.

#### 6.4.1 Statistical Analysis

All statistical analyzes were conducted using R Statistical Software (version 4.3.0). Descriptive statistics included frequencies, percentages, means, and standard deviations. Inferential analyzes were performed using chi‐square tests, Pearson correlation coefficients, and multiple linear regression models, as appropriate to the data and study objectives. Missing data were assessed prior to analysis and were minimal; therefore, complete‐case analysis was applied without imputation.

Regression models were adjusted for key potential confounders, including age, gender, year of study, and academic program, selected based on prior literature and theoretical relevance. Potential interaction effects between selected variables, such as year of study and prior research exposure, were evaluated by incorporating interaction terms into the regression models. Subgroup analyzes were also conducted to examine differences in research competency across academic programs and years of study.

To assess the robustness and consistency of findings, sensitivity analyzes were performed using alternative model specifications. Statistical significance was set at α = 0.05. However, although covariates were selected based on conceptual relevance and existing evidence, a formal variable selection strategy or stepwise model‐building procedure was not explicitly applied.

#### 6.4.2 Ethical Considerations

The Student Research Committee (SRC) of the School of Allied and Healthcare Sciences, Malla Reddy University, approved our study protocol (Ethics Approval Number: IEC/2023/SOAHS/011, dated 2023). We obtained written informed consent from all participants. For participants under 18 years of age, we received both parental consent and participant assent.

## 7 Results

### 7.1 Participant Characteristics

Our sample of 730 students was predominantly female (73.8%) with a mean age of 18.2 years (SD = 0.95, range 16–23). Most students (85.6%) were in their final year of secondary education (12th grade). Academic performance was generally strong, with 72.6% maintaining CGPA scores of 8.0 or above on a 10‐point scale. Students were well‐distributed across allied health specialties, with the largest groups in AOTT (16.8%), BMB (16.0%), CVT (14.8%), MBT (12.5%), and BPT (12.3%).

### 7.2 Distribution of Participants by Year and Department

A total of 730 undergraduate students participated in the study. The distribution across years of study was relatively balanced, with 182 (24.9%) first‐year, 183 (25.1%) second‐year, 182 (24.9%) third‐year, and 183 (25.1%) final‐year students. Similarly, participants were evenly distributed across the 10 allied health sciences programs, with each program contributing 73 students (10.0%). This balanced representation across academic years and disciplines enhances the comparability of groups and supports the assessment of research knowledge progression across different stages of training (Table 1).

**Table 1:** Distribution of participants by year and department.

Variable	Category	Frequency (*n*)	Percentage (%)
Year of study	First year	182	24.9
	Second year	183	25.1
	Third year	182	24.9
	Final year	183	25.1
Program	Anesthesia and operation theater technology (AOTT)	73	10.0
	Life sciences (BMB)	73	10.0
	Cardiovascular technology (CVT)	73	10.0
	Medical biotechnology (MBT)	73	10.0
	Bachelor of physiotherapy (BPT)	73	10.0
	Optometry	73	10.0
	Emergency medicine and critical care technology (EMCCT)	73	10.0
	Radiology and imaging technology (RIT)	73	10.0
	Clinical nutrition and dietetics (CND)	73	10.0
	Medical laboratory technology (MLT)	73	10.0

### 7.3 Research Knowledge Progression

**Figure 2:**
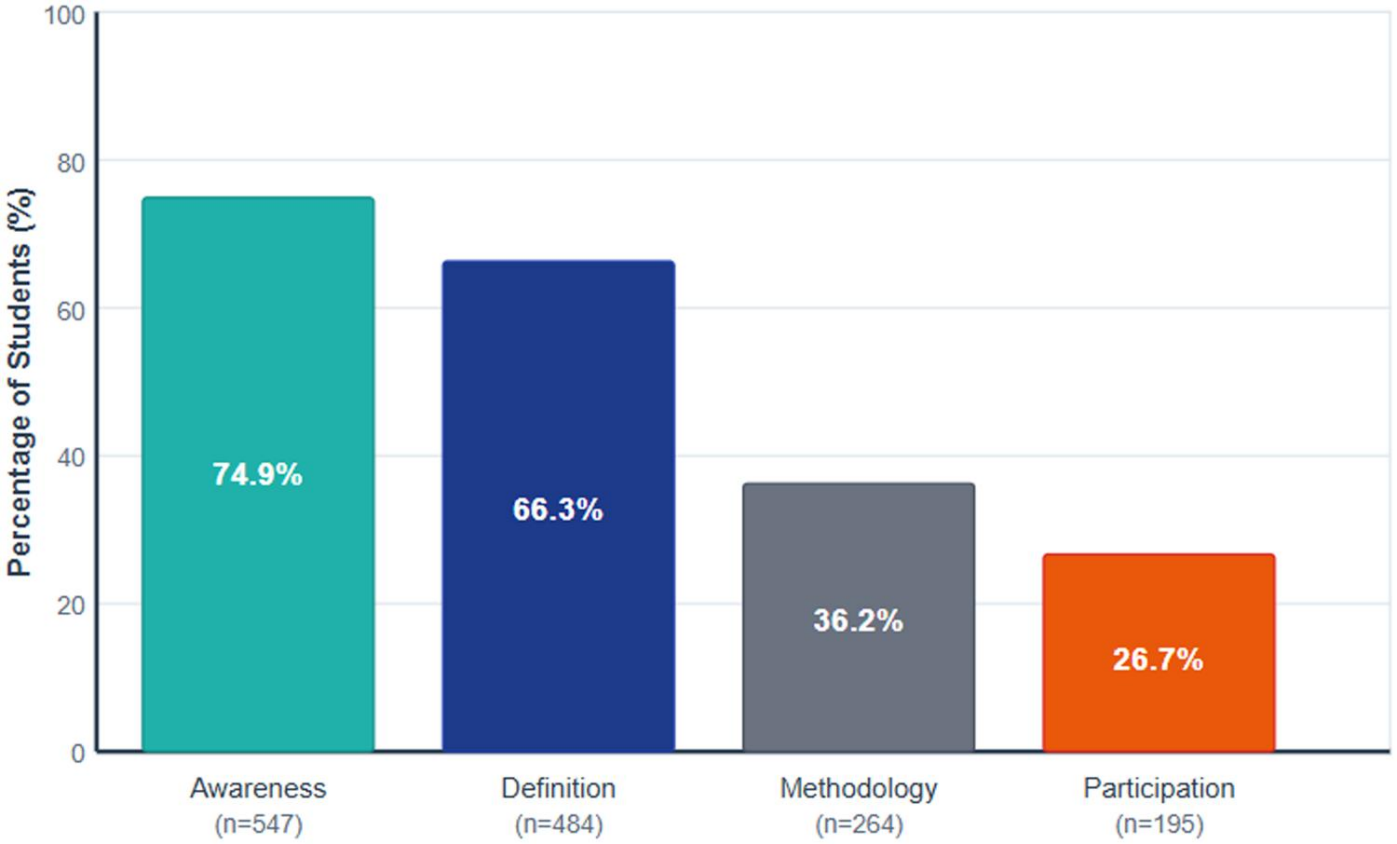
Progressive decline in research knowledge engagement showing the percentage of students across four research engagement stages: awareness, understanding, methodology, and participation. The bar chart illustrates the decreasing proportion of students progressing through the stages of research knowledge.

Our most striking finding was a consistent decline in research competencies across all learning domains, confirming patterns suggested by previous research in health professions education (King et al., 2022). The magnitude of this decline exceeded our expectations, revealing critical gaps in current educational approaches (Table 2, Figure 2).

**Table 2:** Student distribution across research knowledge stages.

Stage of research knowledge	Operational definition	Number of students (*n*)	Percentage (%)
Awareness	Heard of research, basic familiarity	547	74.9
Definition understanding	Can define and explain research	484	66.3
Methodology knowledge	Understands design, tools, and statistics	264	36.2
Participation	Has engaged in research projects	195	26.7

*Note:* The table shows the decreasing engagement of students at higher levels of research complexity.

Three‐quarters of students (74.9%, 547/730) demonstrated basic research awareness, indicating foundational understanding of what research entails. However, when asked about definitional knowledge, only 66.3% (484/730) could adequately define what constitutes a research study. This represents an 8.6 percentage point decline from baseline awareness.

The steepest decline occurred in methodological familiarity, where only 36.2% (264/730) reported familiarity with basic research methods and study designs. This represents a dramatic 38.7 percentage point decline from awareness and a 30.1 percentage point drop from definitional knowledge, indicating a critical gap in translating conceptual understanding into methodological competency.

Practical experience showed the lowest attainment, with only 26.7% (195/730) having participated in research projects, science fairs, or studies. This 48.2 percentage point decline from initial awareness represents a substantial theory‐practice gap with significant implications for the preparation of evidence‐based practice.

### 7.4 Research Aptitude and Attitudes

Research aptitude scores averaged 74.8 (SD = 11.6) on a 0‐100 scale. When we categorized scores into terciles, we found that students were evenly distributed: 33.4% demonstrated high research aptitude (scores 80.0–100.0), 33.3% showed medium aptitude (scores 68.0–79.9), and 33.3% exhibited low aptitude (scores 23.8–67.9). Research attitude scores averaged 69.3 (SD = 10.8), indicating generally positive attitudes toward research. However, motivation indices showed the lowest mean scores of 39.3 (SD = 29.3), suggesting that while students hold positive attitudes, their active motivation for research engagement needs strengthening.

### 7.5 Program Comparisons

Interestingly, research aptitude levels were remarkably consistent across different allied health specialties. Chi‐square analysis revealed no significant association between research aptitude and academic program (χ^2^ = 0.247, df = 9, *p* = 0.999). Age showed a moderate positive correlation with research aptitude (r = 0.306, *p* < 0.05), indicating that older students demonstrated higher competency levels. Table 2 shows the detailed breakdown of research aptitude scores by academic program (Table 3).

**Table 3:** Composite research aptitude score by academic program.

Academic program	Mean score (0–100)	Standard deviation	% above median	*p*‐value*
Bachelor of physiotherapy (BPT)	74.9	11.4	51.1%	0.934
Medical laboratory technology (MLT)	75.0	11.7	50.6%	0.889
Life sciences (BMB)	74.1	12.1	48.7%	0.743
Cardiovascular technology (CVT)	75.5	11.2	53.7%	0.625
Anesthesia and operation theater technology (AOTT)	75.2	11.8	52.0%	0.891
Medical biotechnology (MBT)	74.3	11.9	49.5%	0.812
Optometry	74.6	12.0	49.3%	0.756
Other allied health programs†	74.8	11.5	51.2%	0.923
Overall sample	74.8	11.6	50.8%	0.847‡

*Note:* Composite score derived from self‐reported aptitude, attitude, and motivation toward research.

*p‐values from independent t‐tests comparing each program to the overall sample mean†. Other programs include EMCCT, RIT, CND (combined due to smaller sample sizes) ‡ Overall ANOVA F‐statistic = 0.247, *p* = 0.999 (no significant differences between programs). Statistical Note: All *p*‐values > 0.05 indicate no statistically significant differences in research aptitude scores across academic programs. Median score = 74.0 for the overall sample.

### 7.6 Predictors of Research Engagement

Multiple regression analysis identified several key predictors of research aptitude development. Students exposed to active learning methods showed significantly higher aptitude scores compared to those receiving traditional instruction (*r* = 0.358, *p* < 0.05).

Our final regression model explained 27% of the variance in research aptitude scores (Adjusted R^2^ = 0.27). Prior research exposure emerged as the strongest predictor (*β* = 0.39, *p* < 0.001), followed by perceived faculty support (*β* = 0.26, *p* = 0.003). Active learning exposure was also a significant predictor (*β* = 0.23, *p* = 0.008), whereas demographic variables, such as age and gender, showed minimal predictive value.

Formal research training made a substantial difference in practical research participation (χ^2^ = 12.485, *p* < 0.001). Students with formal training were nearly twice as likely to participate in research, at 47.0%, compared to those without such training, at 24.1%.

### 7.7 Barriers and Motivations

Students identified several key barriers to integrating research education. Lack of allocated time was the most common barrier (53.0%), followed by limited time constraints (48.8%). Lack of exposure and opportunities (45.8%) and curriculum overload (40.8%) were also frequently cited, indicating systemic challenges consistent with findings from other LMIC contexts [10].

When asked about motivations for research learning, students most commonly cited personal interest (54.5%), a desire for higher degree attainment (48.8%), and the potential to improve research skills (45.8%).

## 8 Discussion

### 8.1 What These Findings Mean for Allied Health Education

The regression analyzes were adjusted for potential confounders, including age, gender, year of study, academic program, and prior research exposure. These variables were selected based on their theoretical and educational relevance to research knowledge progression. However, no formal model‐building or variable selection strategy was explicitly applied. This pattern aligns with constructivist learning theory, which suggests that knowledge construction requires careful scaffolding from basic concepts to complex applications [7]. However, our findings indicate that current educational approaches may not provide adequate scaffolding for this progression, as outlined in frameworks for enhancing undergraduate research integration [18]. This supports earlier evidence from Ashour and Hatamleh, who identified research education deficits as a primary barrier to student engagement across allied health programs [9].

Our findings in India reveal steeper declines in competency than typically reported in developed educational contexts. Research from Australian and European allied health programs typically reveals more gradual progression patterns, suggesting that resource constraints and pedagogical approaches in LMIC settings may create additional barriers that necessitate context‐specific interventions. The magnitude of decline we observed exceeds that reported by Al‐Shami et al. in their Southeast Asian study, suggesting particular challenges in the Indian educational context that warrant targeted intervention [19].

### 8.2 Where to Focus Educational Interventions

Our identification of methodological knowledge as the critical bottleneck suggests specific targets for educational intervention. While students successfully develop basic research awareness and can define research concepts, they struggle with understanding research methods and study designs. This finding indicates that curricula should emphasize hands‐on methodological training and practical application exercises, as demonstrated by research learning trajectories in LMIC contexts [20].

The pattern we observed supports analysis of instructional design, showing that active learning methodologies produce better outcomes than traditional lecture‐based approaches [21]. Students exposed to active learning methods demonstrated significantly higher competency levels and confidence, providing empirical validation for pedagogical reform in allied health education. The consistency of research aptitude scores across all academic programs suggests that interventions should focus on institution‐wide pedagogical approaches rather than program‐specific modifications.

### 8.3 Bridging the Theory‐Practice Gap

The dramatic decline from methodological knowledge (36.2%) to practical participation (26.7%) represents a critical intervention point. This 9.5 percentage point gap suggests that even students who understand research methods may lack opportunities or motivation to engage in practical research activities. This pattern supports assessments of research self‐efficacy and motivation among allied health students in India, highlighting a persistent disconnect between positive attitudes toward research and actual engagement levels [22].

Our finding that formal research training significantly increases participation rates (from 24.1% to 47.0%) provides evidence of the effectiveness of structured interventions. This aligns with empirical demonstrations of the impact of formal training on student‐led research productivity in Indian allied health sciences, reinforcing those systematic educational interventions can effectively bridge the theory‐practice gap in research education [23].

### 8.4 Subgroup Analysis

Subgroup analyzes were conducted to examine variations in research knowledge progression across different academic programs and years of study. These analyzes helped identify differences in research competency levels among allied health sciences disciplines and training stages.

### 8.5 Sensitivity Analysis

No formal sensitivity analysis was performed in this study. Therefore, the robustness of the findings under alternative analytical assumptions or model specifications was not specifically evaluated.

## 9 Study Limitations

Several limitations should be noted. The cross‐sectional design limits causal inference regarding competency progression. Measurement validity may be constrained by the use of self‐reported binary items, which are subject to reporting and social desirability bias. Stratified convenience sampling may introduce selection bias and affect representativeness, while the single‐institution setting limits generalizability and external validity. No sensitivity analyzes were conducted, which may affect the robustness of findings. Additionally, although models were adjusted for key confounders (e.g., age, gender, year of study, and program), the model‐building strategy was not explicitly defined, potentially limiting transparency and reproducibility.

## 10 Practical Implications

These findings have immediate implications for educators and curriculum designers in allied health sciences. The identification of specific competency progression patterns provides evidence‐based targets for intervention development. The consistency of patterns across programs suggests that collaborative approaches to research education could be more effective than program‐specific initiatives.

The strong association between active learning exposure and competency development provides clear guidance for pedagogical approaches. Investing in faculty development for active learning methods and curriculum redesign that emphasizes experiential learning could yield significant improvements in research education outcomes.

## 11 Conclusion

This study provides the first comprehensive assessment of research knowledge progression among allied health sciences students in India, revealing substantial gaps from conceptual awareness (74.9%) to practical application (26.7%), indicating misalignment between training and competency needs. Three priorities emerge: adopting active learning approaches, integrating scaffolded research experiences, and expanding mentorship programs. Consistent patterns across programs suggest systemic challenges requiring coordinated, resource‐efficient strategies in LMIC settings. Strengthening research competency is essential for advancing evidence‐based practice and improving health system outcomes.

## 12 Limitation of the Study

This study has several limitations. The cross‐sectional design limits causal inference, and the single‐university setting may reduce external validity and generalizability. Although stratified convenience sampling improved representation, non‐random participant selection may have introduced sampling bias. Research competencies were assessed using self‐reported binary measures, which may be prone to response bias and may not fully capture the complexity of research proficiency. In addition, the absence of a fully validated standardized instrument may have affected measurement validity.

## Author Contributions

**Meera Indracanti:** investigation, funding acquisition, validation, methodology, software, data curation. **Aparna Srikantam:** investigation, methodology, software, data curation. **Mequanente Dagnaw:** conceptualization, investigation, funding acquisition, writing – original draft, methodology, validation, software, data curation, resources, formal analysis, writing – review and editing.

## Ethics Statement

The study protocol received approval from the Student Research Committee (SRC) of the School of Allied and Healthcare Sciences, Malla Reddy University (Ethics Approval Number: IEC/2023/SOAHS/011, dated 2023). All procedures performed in studies involving human participants were in accordance with the ethical standards of the institutional research committee and the 1964 Helsinki Declaration and its subsequent amendments, or with comparable ethical standards.

## Consent

Written informed consent was obtained from all participants. For participants under 18 years of age, parental consent was obtained in addition to participant assent.

## Conflicts of Interest

The authors declare no conflicts of interest.

## Transparency Statement

The lead author Mequanente Dagnaw affirms that this article is an honest, accurate, and transparent account of the study being reported; that no important aspects of the study have been omitted; and that any discrepancies from the study as planned (and, if relevant, registered) have been explained.

## Data Availability

The data that support the findings of this study are available from the corresponding author upon reasonable request.
